# Automated Adjustment of PPE Masks Using IoT Sensor Fusion

**DOI:** 10.3390/s23031711

**Published:** 2023-02-03

**Authors:** Ashish Manchanda, Kevin Lee, Gillud David Poznanski, Alireza Hassani

**Affiliations:** 1 School of Information Technology, Deakin University, Geelong, VIC 3220, Australia; 2 Car Advance, Ivanhoe, VIC 3079, Australia

**Keywords:** PPE, smart masks, automatic adjustment

## Abstract

The COVID-19 pandemic has led to a dramatic increase in the use of PPE by the general public as well as health professionals. Scientists and health organizations have developed measures to protect people and minimize the catastrophic outcomes of COVID, including social distancing, frequent and periodic sanitizing, vaccinations, protective coverings, and face masks. During this time, the usage of protective face masks has increased dramatically. A mask only provides full safety to the user if it is a proper fit on their face. The aim of this paper is to automatically analyze and improve the fit of a face mask using IoT sensors. This paper describes the creation of a 3D-printed smart face mask that uses sensors to determine the current mask fit and then automatically tightens mask straps. This is evaluated using adjustment response time and the quality of fit achieved using the automatic adjustment approach with a range of sensor types.

## 1. **Introduction**

Face masks are one of the most important components of effective PPE and this effectiveness depends on many factors that include the material and structure of the mask. The risks of viral transmissions and contamination are higher in healthcare personnel [1]. Experiments testing masks against saline aerosol dust have demonstrated that dust masks were only 6.1% efficient, bandanas 11.3% efficient, and surgical masks 33.3% efficient, whereas N95 masks were the most effective out of these four with their efficiency rate being 89.6% [2].

Certified masks are designed in such a way that they do not hinder the ability of the frontline workers. The reason most of the medically certified masks have tight straps is to provide the user with a complete fit on the face. According to research, a user has the best protection from viruses if the mask is covering the full face including edges, i.e., fits on the face. However, that fit is not monitored on a constant basis, and thus it becomes difficult to analyze when the mask is losing the original position of fit. After sensing the displacement, there is a need to tighten the mask and achieve the fit again.

The aims of this paper are to address the issue of achieving a good fit of the mask through the use of a sensor-enhanced mask and automatic adjustment mechanism. The contributions of the work are: (i) a thorough literature research and market analysis to identify flaws in existing products and designs; (ii) the design and building of a prototype smart mask solution which utilizes various sensors and automatically adjustable strap mechanisms; (iii) an evaluation of the effectiveness of the automatic adjustment approach with a range of sensors.

The remainder of the paper is as follows. Section 2 reviews the literature. Section 3 describes the design and implementation of the prototype automatically adjusting mask. Section 4 evaluates the approach under a variety of scenarios. Section 5 presents a discussion of the effectiveness of the solution. Finally, Section 6 presents conclusions and discusses future work.

## 2. Literature Review

### 2.1. Personal Protective Equipment (PPE)

With the ongoing evolution of technology, the design structure of the PPE evolves every year with the goal of making it more compatible, easier to use, sustainable, and ’Smart,’ which means giving other capabilities other than merely filtering the air, such as tracking human engagement. [3]. Cross-contamination caused by the incorrect removal and fitting of masks and gowns was one of the most serious hazards noticed in the healthcare profession. As a result, PPE must be used in conjunction with air filtration which lowers the chances of infection. [4]. In recent years, there has been an increase in the adoption of smart solutions for healthcare processes. Multiple steps have been taken that have been proven beneficial. [5]. Various research supports that it is possible to use IoT-integrated PPE which has proven to fight most effectively against viruses with the extra benefit of inbuilt health monitoring [2,6,7,8].

### 2.2. Issues with PPE

Despite its many advantages and benefits, personal protective equipment (PPE) has some drawbacks. These concerns are minor when weighed against the benefits of PPE, which has no substitute as of now [5,9]. The following are the most prevalent issues with PPE.

- Excessive sweating 100%—wearing the PPE for a long time [9].
- Fogging of goggles, spectacles, or face shields 88%—air trapped between the glasses and the wearer’s face is hotter and more humid because the worker’s skin is hotter and perspiring, increasing the likelihood of fog [5].
- Suffocation 83% and breathlessness 61% [9].
- Fatigue 75% and headache due to prolonged use 28% [5].

These disadvantages can be overcome with contemporary technology. To make the PPE operate in accordance with the ’sense, think, and act’ paradigm, progress on the operating conditions should be made during the early design stage of product design, as requirements are sometimes missed when incorporating smartness into PPE [9,10,11].

### 2.3. Adaptive Fit of Masks

PPE face masks must be in close contact with the skin to provide better protection from contamination [12]. Wearing the mask generally leaves infraorbital skin open to the surroundings, leading to airflow from inside out and vice versa without filtration. Therefore it becomes essential that the mask must be in close contact with the skin at all times while conducting activities like walking, speaking, etc. Although N95 masks are suitable barriers against airborne infections like COVID-19, poorly fitted masks can have significant leaks around the face, reducing their efficacy and increasing the risk of infection [13,14].

### 2.4. Current State of Mask Fitness

Methods to improve the fitness of the mask include tightening the knot of the mask straps [15], using lanyard straps, elastic braces of the mask, and wearing double masks. Research shows that different masks offer different types of fit depending upon the face structure of the user. When comparing the different sets of masks such as N95, KN95, surgical, and cloth on the basis of comparing the fitness/protection using the respirator test the results were positive. Despite having high filtration in KN95 and N95, they offer a similar level of protection to that of fabric and surgical masks because of lower and improper fit. This shows that one particular type of mask is not fit for the general public because of poor fit. A huge impact is observed on minor facial differences or the structure of the face mask [16].

There are two accepted methods for assessing the fitness of a mask [17]. The first is a quantitative fit test that uses a machine to check for leakage around the face seal. This results in a ’fit factor’ number of around 100 for half-mask respirators, and between 500 and 1000 for full facepiece respirators. The second method of fit test is qualitative fit testing, which asks the user to detect tastes or smells through the mask. A good seal is achieved when no external smells are detected.

There are methods to improve the fit of face masks. Tape can be used to cover the edges of masks to improve the seal [17]. It also states that the high filtration material masks used with proper fit provide the best protection to the user. A modern approach to maximizing mask fitness is to use a 3D-printed customized fit mask that is produced based on the face structure of the user and is a proper fit for the face [18].

These methods, providing a better fit of the mask, are secure and tested through different equipment such as respirator fit testing. The only drawback is that after achieving a better fit, comfort is lost to the user, which is again an important factor. In a nutshell, better filtration, proper fitting of the mask, and comfort cannot be achieved simultaneously; out of three, one is compromised at all times [13,14,15,16,17]. This shows the high need for a technology that can inculcate all three behaviors in one.

### 2.5. New Modification in the Mask to Achieve Fitness

There are a variety of ways to improve the fitness of face masks by making small modifications to the way the user wears the mask. The authors of [13] performed experiments to highlight to effectiveness of different approaches, summarized as follows.

1. Earloops crossed: this can be achieved by tightening the knot to each string of the mask to achieve the maximum fit.
2. Earloop strap: this can be achieved by using a strap in which straps of the mask can be tucked and varied at different lengths.
3. Earloop toggle: this can be achieved using the toggles—the straps of the masks are passed through these toggles, which in turn help to shorten the length of the string.
4. Mask brace: this can be achieved by using the external rubber/plastic material which fits on the top of the face mask and provides extra fit and tightness to the user.

Of these approaches, the mask brace provides the best performance when evaluated using a respirator test fitting. A good mask fitting can provide similar performance as that of double masking or a 3-ply mask [13]. The mask brace covers the edges of the mask so that it remains in contact with the skin [19].

More advanced approaches to maintaining mask fitness include the harmonic sensing technique that monitors the movement of the masks [20]. This approach uses a radio signal to transmit and receive back a harmonic frequency which is used to detect coughs and the fit of the mask. The fit here only determines the covering of the face, not the external edges. FaceBit’s smart sensor technology retrofits masks with a pressure sensor that measures leaks to indicate how well the mask is fitted [21]. That approach focused on N95 or KN95 masks because their mask structure holds the skin firmly and tightly so any displacement can be easily detected by the sensor.

### 2.6. Methods for Adaptive Fitting in Wearable Technologies

Adaptive fitting technologies can be used to create the automatic adjustment of clothing. Approaches include using dual adaptive adjustment for customized garment plans and using a soft elastomer tube made from polydimethylsiloxane as a u-shaped tube in the shoes for bright fitting. Apple is researching using a Nitinol mixture of Nickel and titanium that will contract and expand based on electrical signals with a fluid- and gas-filled bladder. Nike has also developed Nike Adapt BB shoes that automatically tighten the laces by pressing a button. However, there is no such innovation for masks that can adjust the fitting based on the displacement of the mask on the skin while performing activities [22,23,24,25].

### 2.7. Current State of Fitness of PPE Masks

N95 masks have been demonstrated to be the most effective in protecting against widespread diseases such as COVID-19 [2]. Despite efforts by researchers to create a mask that not only reduces viral transmission but also addresses issues related to difficulty in breathing, air purification, and a more comfortable design, there remains a significant gap in regards to the mask’s consistent fit. This is a crucial issue for the mask’s primary function [5,26]. Sensors can be utilized to determine the lifespan of the mask by collecting environmental data using IoT sensors, however, this does not address the adjustment or tightening of the mask, which is a crucial factor in minimizing contamination [27].

Table 1 presents a review of the state of the art in smart masks. The table contrasts the key components and behaviors of the masks. All of the masks fit comfortably. FaceBit is not trendy, whereas Project Hazel does not provide cross-contamination but is lighter than other masks. Although Forcit, Air Pop, and FaceBit warn users of filter changes, which is another component in providing additional protection to the user, none of them focused on adjusting fitness or tightness [28]. To discover a method for modifying the mask’s adaptive fitness and tightness, certain sensors and actuators that can feel the resilience between the mask and the user’s skin and change the mask appropriately are required. This undoubtedly differs and is unique to each individual based on their health and circumstances [29].

### 2.8. Smart PPE and the Internet of Things (IoT)

Smart PPE and smart face mask approaches follow advances in embedded systems and Internet of things technologies. The IoT allows the creation of smart approaches to improve working environments [30]. Initially, smart PPE is single-use, closed loop, but will follow trends in smart healthcare and be integrated with other smart PPE and IoT technologies to be part of holistic smart healthcare solutions. IoT applications are built using a variety of frameworks and platforms [31,32] that support the integration of hardware, software, and people. For smart PPE to be a part of this, the device must be connected to the Internet and data must flow into the Cloud. Once these data are in the Cloud, they can be processed for physiological characteristics [33,34]. This paper proposes a prototype smart PPE device that can be connected as an IoT device to the Cloud to provide rich data.

### 2.9. Statistical Analysis of the Current State of the Mask

The impact of wearing a mask on an individual’s performance during various activities is inconclusive and may vary depending on the nature of the activity. For instance, the physical exertion will be higher when wearing a mask while performing strenuous physical activity [35]. According to author Keely A. Shaw, wearing a mask during physical activity does not significantly impact the performance of the activity, but may have slight effects on physiological performance [36]. Both surgical and N95 masks have been evaluated through systematic approaches and meta-analyses, with findings indicating that the rate of exertion increase was higher for N95 masks.

To effectively protect an individual, a mask must fit and seal tightly to the face, which is the rationale behind performing respirator fit testing for healthcare workers. The respirator fit test improves the fit of the mask on the face, but to maintain this fit, a smart sensing technology that continuously monitors the position of the mask is needed. Multiple IoT sensors, such as pressure sensors, light-intensity sensors, and temperature–humidity sensors can be used to measure the fit of the mask. Additionally, an actuator is necessary to adjust the mask’s fit if it becomes altered.

Another approach that can be taken is using machine learning and neural networks to determine the fit of the mask on the face. In this approach, images of individuals wearing different mask structures are fed into a machine learning model with a convolutional neural network [37]. This approach can serve as an alternative to the respirator fit testing and qualitative fit testing used by professional healthcare workers.

## 3. Design of an Auto-Tightening Smart Mask

**Figure 1:**
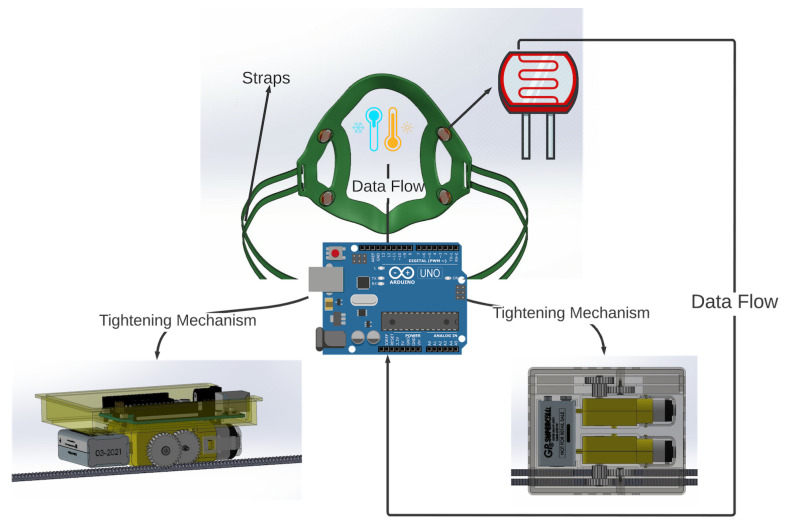
Prototype of the 3D smart mask.

To solve the problem of incorrect mask fit, this section presents the design of a prototype smart face mask that can automatically adjust itself for optimal fit, presented in Figure 1. It consists of a mask brace embedded with four light sensors, connected with the four straps, and the other end of the straps are connected to the motor and a micro-controller for processing environmental data and controlling the motors for mask tightening. The working principle of this design collects data from sensors and uses an algorithm to observe the current mask fit based on light intensity and controls the rotation of a servo motor.

**Figure 2:**
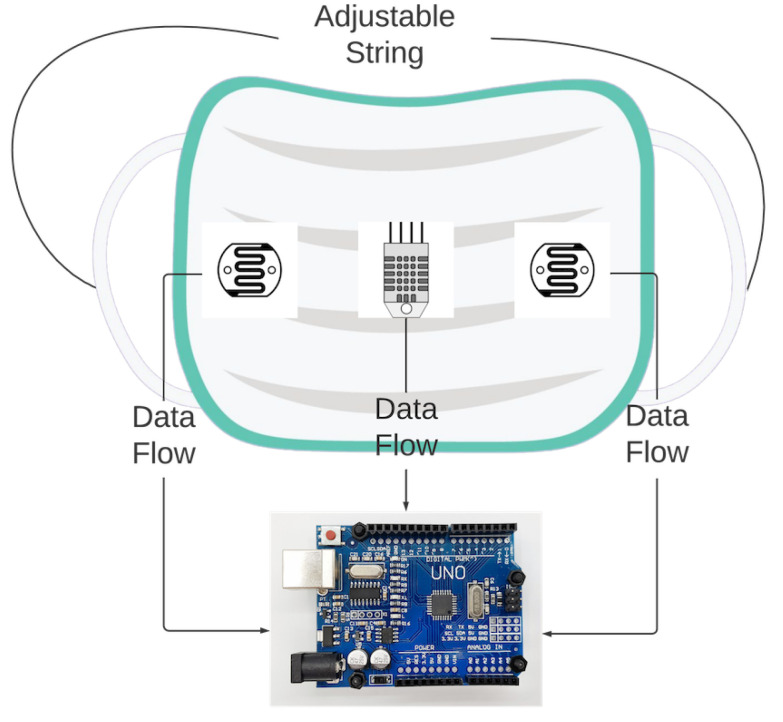
Prototype of the smart mask for Stage 1 Process.

To create an effective design for the auto-tightening mask, a two-stage process was chosen. Firstly, a series of experiments was performed to retrofit sensors to three distinct types of commercially available PPE masks. Through those experiments, the best positions to retrofit the sensors and the best masks were determined for the ideal tightness and comfort level of the user. Secondly, a 3D-printed mask brace that enabled the mounting of the sensors was designed, with microcontrollers and motors/gears to perform the tightening mechanism. The results from the first experiments were used to inform the type of material used, the calibration of the sensors, and the auto-tightening algorithm. The prototype design for the first stage is illustrated in Figure 2, which consists of a microcontroller, two light-dependent resistor (LDR) sensors, one temperature/humidity sensor, and an adjustable strap connected to the mask. LDR sensors are used to determine the intensity of the light inside the mask. To achieve reliable sensor accuracy for this mask, two LDR sensors were used, one on each side of the mask. A single temperature sensor detects the temperature and humidity of the user when the setup is worn. Data collected from the temperature/humidity sensor and the LDR sensors are used to measure the current mask fit and the rate of change of the length of the mask strap. Section 2.9 states the reason for utilizing temperature and humidity sensors so that data can be gathered from the breathing of an individual and based on that data, an analysis can be made to track the health of a person.

**Figure 3:**
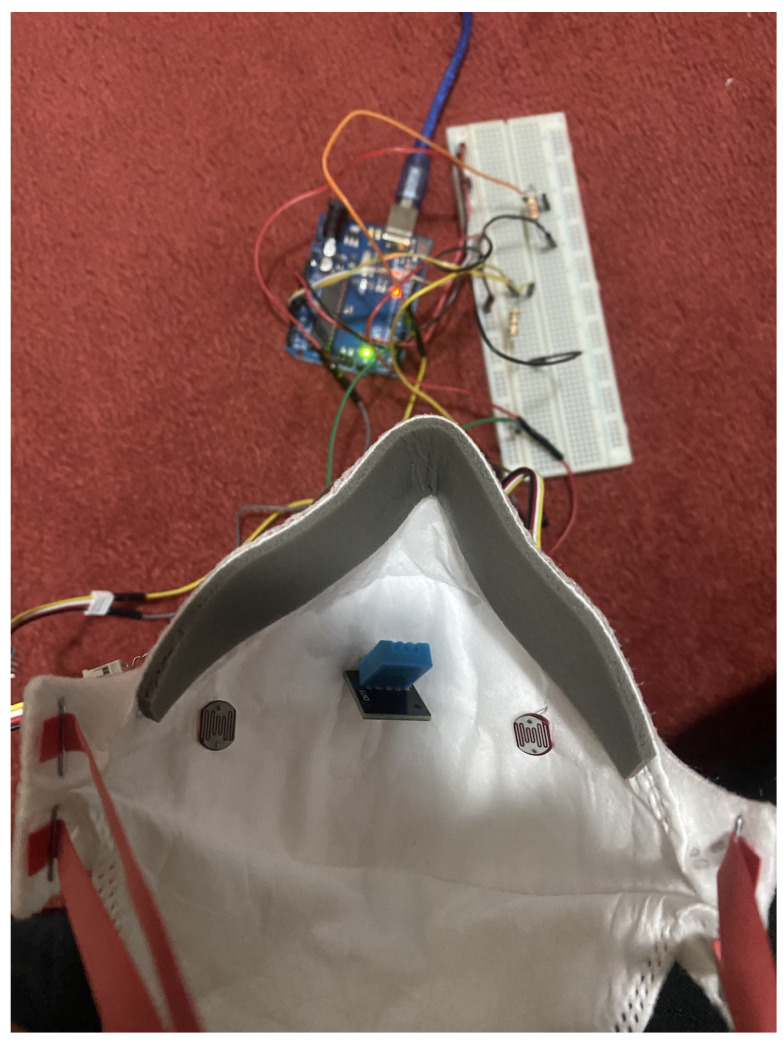
Set up of sensors inside the mask for Stage 1 process.

Figure 3 illustrates the configuration of the first prototype mask created to evaluate sensor placement. The initial investigation revealed that it is vital to cover the whole surface of the mask as well as the most sensitive parts to air/light. It was determined that the DHT11 temperature/humidity sensor, which is used to detect temperature and humidity within the mask, is best placed in front of the mouth at the mask’s center in order to achieve accurate sensor readings. This is because the temperature and humidity levels vary with respect to the position of the sensor within the mask and if it is touching the skin. The readings are mostly influenced if the user breathes in/out and the sensor detects it immediately. An Arduino UNO R3 was used to collect data from the sensors attached to the mask and transfer this data to a computer for analysis.

**Figure 4:**
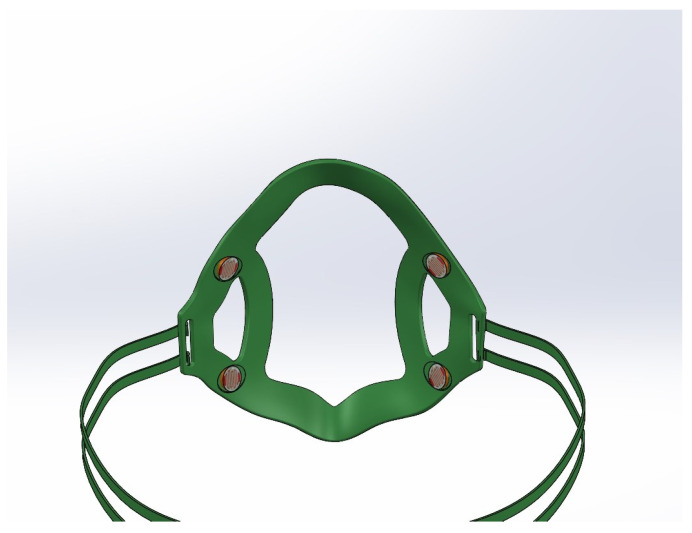
Mask brace with LDR sensors.

Every person’s face structure is different which makes it difficult to achieve and then attain the best fit of the mask. Due to this, those individuals most at risk have customized masks made which are designed to better fit their face. It is difficult to build a mask that is a proper fit for any individual. This issue highlights the need for a technical solution to improve the fit of the mask on the face. To achieve this, a prototype ’Mask Brace’ in Dassault Systèmes SOLIDWORKS CAD design software as seen in Figure 4 is modeled. This includes locations for four sensors: two sensors on the left and two on the right. The upper two sensors are connected to the upper two straps of the brace whereas the lower ones are connected to the lower straps. The shape and design of the brace are the motivation from the structure of the KN95 mask, which firmly holds on the face and provides a cup-like structure. Two gaps are made near the sensor positions to reduce the error and increase the precision of sensor readings. The concept is to engage the motor if the LDR sensors sense any light inside or near the edges of the mask until the intensity of light is limited.

**Figure 5:**
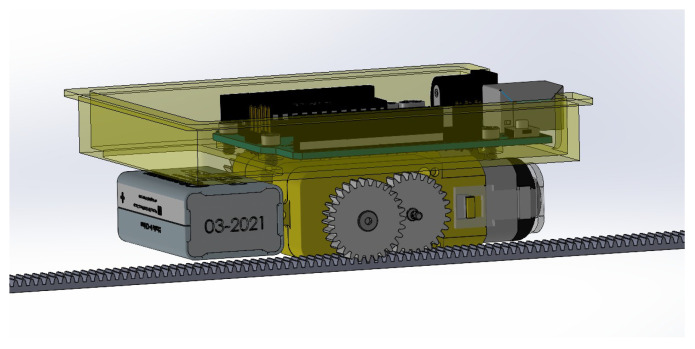
3D design of Arduino mounted on the gearbox.

The mask brace is tightened by using data from the sensors attached. Data are sent to the microcontroller mounted on the top of the gearbox as shown in Figure 5 which is connected to the brace through straps. An algorithm controls the rotational power of the servo motor based on the values of light from the sensors mounted in the mask. Gears are attached to the motors which start rotating when light is detected. The straps are retrofitted with the gears and another end is attached to the mask brace. Thus, when gears are rotated, a pull mechanism is generated which pulls the straps, and as a result the mask brace is pulled towards the face, tightening it.

**Figure 6:**
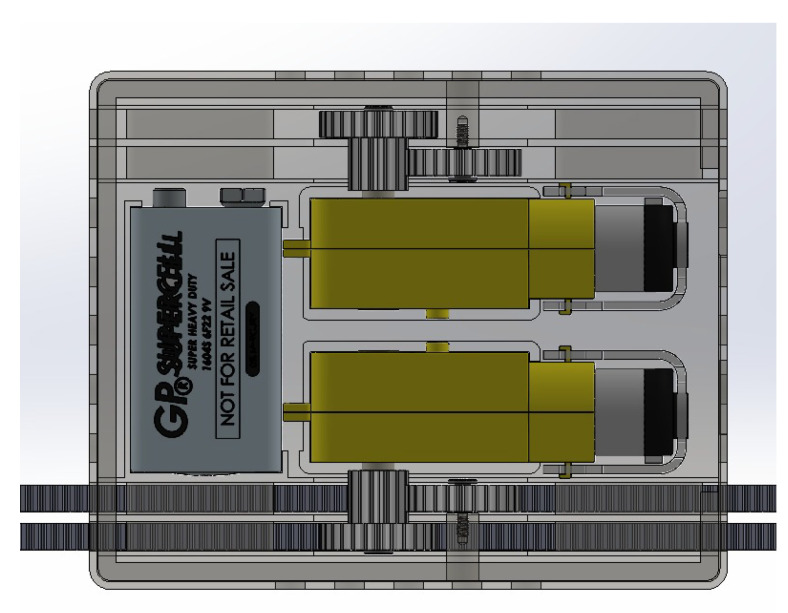
3D design of gearbox with DC motors and straps.

The Arduino microcontroller is connected to two DC motors which are connected to two gears as shown in Figure 6 that rotate to tighten the strap. The Arduino is powered with a nine-volt battery fitted inside the gearbox. One DC motor can only produce enough power to pull the strap on either side of the mask brace. Two gears are used to provide enough stress and shear strain so that when the pull mechanism takes place the strap is held instead of breaking out. If the motors start rotating simultaneously more tension is generated which is handled by the two gears which are held tight with the screws.

**Figure 7:**
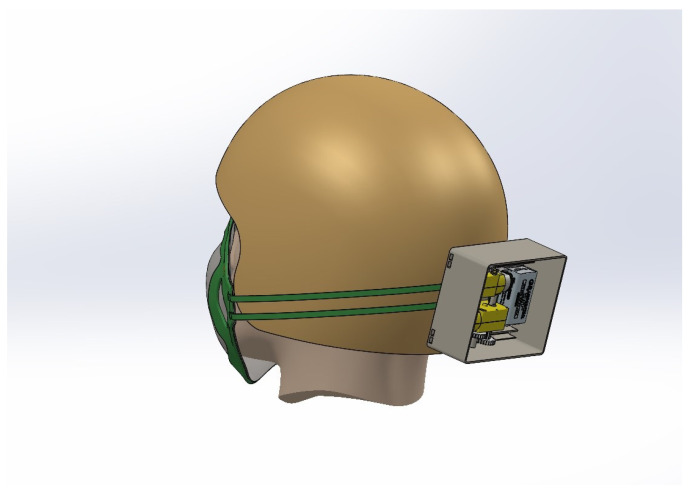
3D design of person wearing mask brace.

To hold all the necessary devices such as motors, gears, and batteries, a control box is needed that will be comfortable for wearing and will hold strong. Thus, when the person wears the mask, it will look as in Figure 7, which was worn by the author while conducting the experiment. The mask brace was built for the technical proof of concept not for human trial so no ethics approval was needed. The DHT11 temperature and humidity sensor is attached to the middle of the mask brace on the mask filter, which captures the temperature and humidity data inside the mask, which then is compared to the temperature and humidity outside the mask. The reason behind doing this is to add another factor that can validate and support the theorem of mask tightening using the LDR sensor.

**Figure 8:**
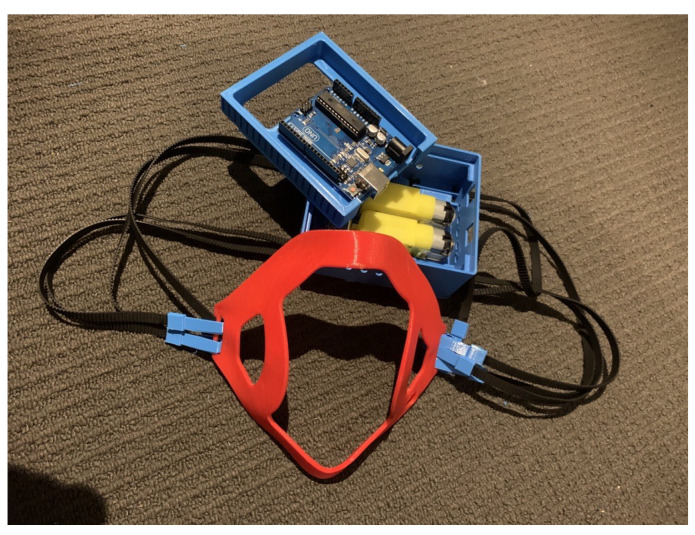
Prototype 3D-printed automatically adjusting smart mask.

The position of the sensors is at four different locations and away from the skin of the user due to the structure of the mask brace. The LDR sensors have a circular field of view, which means that in order for light hitting the surface of the sensor to be detected, it needs to provide enough room for light. There are four straps connected to the mask brace: two at the top and two at the bottom. The tightening of the mask brace happens through tightening the straps. When the sensor senses that the light gears start rotating, one gear rotates clockwise, pulling the string towards it whereas the other anti-clockwise gear pushes it away. A prototype of the 3D-printed mask with sensor mounting, control box, and automatically adjustment mechanism can be seen in Figure 8.

## 4. Evaluation

In this section, an evaluation is performed of the automatically adjusting mask design proposed in Section 3. This evaluation is divided into two subsections. Section 4.1 first presents an evaluation of the use of light in three different commercially available masks to determine mask usage feasibility. Section 4.2 investigated the use of temperature and humidity with these three mask types. Section 4.3 presents an evaluation of the mechanism for automatically adjusting and tightening the mask.

### 4.1. Evaluating Light Sensors with Commercially Available Masks

The aim of this experiment was to measure the variation of light intensity with the strap at different lengths and compare the observation between three distinct masks. There are a variety of environmental factors through which the quality of the fit of the mask can be determined, including humidity, temperature, light, and amount of O_2_ or CO_2_. These factors indicate a physical property; e.g., for light, if no light gets inside the mask, then it can be assumed that a reasonable fit is achieved. For this reason, the intensity of light sensed by light sensors plays a prominent role in deciding the fit of the mask.

**Figure 9:**
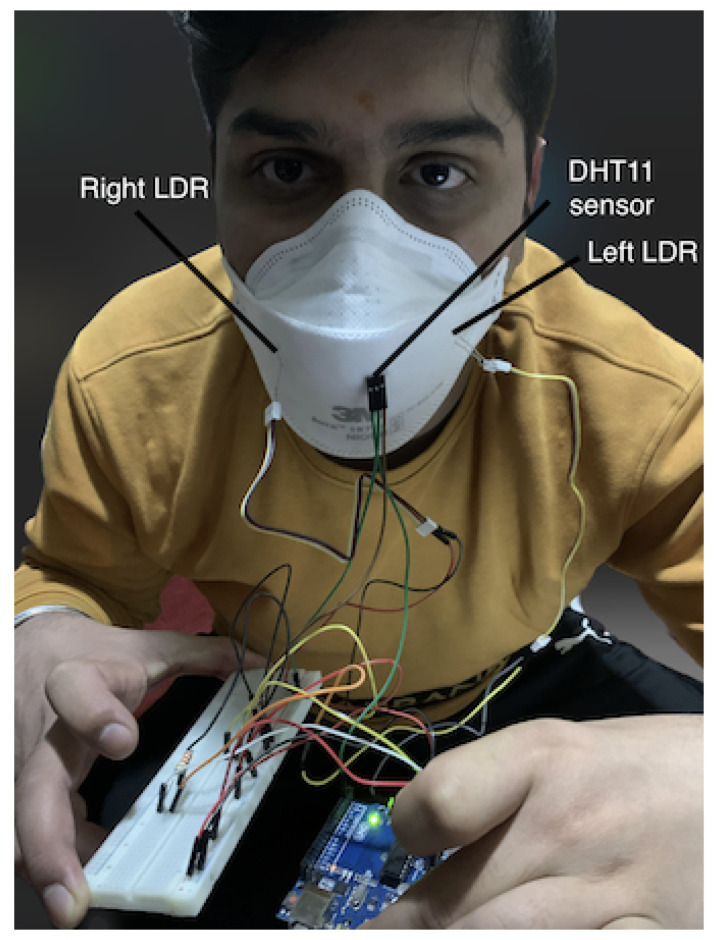
Setup worn by user.

This experiment sought to investigate the feasibility of utilizing light-intensity sensors to determine mask fit quality with commercially available masks. To measure the sensor values at various fit levels, a setup was constructed which allowed for the testing of different masks, as depicted in Figure 9. The experiments were conducted by collecting the intensity of light captured by the light intensity sensors as the adjustable strap length was varied. The setup worn by the participant consisted of a 19.25 cm adjustable strap attached to the mask. The sensors were connected to the Arduino UNO microcontroller through jumper wires. The light intensity sensors were positioned on each side of the mask facing the user’s face, allowing for the detection of light and air/moisture breaches.

**Figure 10:**
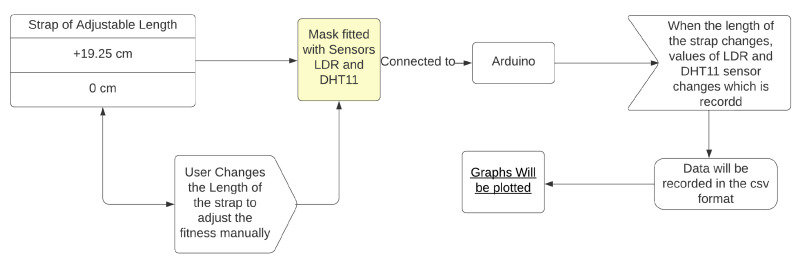
Design and plotting of data.

The results of each of the following experiments were documented by repeating them ten times, varying the strap length from 19.25 cm to 0 cm, and averaging the results. The design of the experiment is illustrated in Figure 10. It states the length of the strap which varied, and which sensors being retrofitted with the mask were connected to the Arduino. Then, sense, think, act paradigm graphs were created from the recorded data. At each point of decrement in the length of the strap the user perception of tightness was also recorded to understand the correlation between strap length, user perception of tightness, and light intensity.

The experiment was carried out on three masks made of different materials. The following masks are used:

- Surgical Mask | Polyester
- Fabric Mask | Cotton
- KN95 Mask | Polypropylene Plastic Polymer

For the first experiment, a surgical mask was used, and the length of the strap was set to its most loose setting. Over time, this was tightened by 2.25 cm each step until it reached its tightest setting. Table 2 contains the results of the experiment with the light intensity for the left and right sensors recorded at each tightness step. The range of the light sensor varied from 0 to 1024, where 0 indicates no light and 1024 indicates the maximum light sensed by the sensor. At the loosest setting of 19.25 cm, the light intensities were measured at 9 for the left side and 8 for the right side of the mask. The data show, as the experiment progressed and the mask was tightened, that the light intensity decreased over time until it reached the lowest value possible by the sensor. This demonstrates that light sensors can be used to determine the tightness of a mask.

The data indicate that the intensity of light detected by LDR sensors with a surgical mask is directly proportional to the length of the strap, implying that as the length reduces, so does the distance between the mask and the user’s face. When the length of the strap approaches 0 cm, the intensity of both sensors drops to 1, indicating that the mask is closest to the face without any gaps. According to the user, the face mask is now too tight. The variation for both sensors left and right is similar in the beginning but changes in the middle when the strap length is changed to 6.75 cm. It is suspected that in the initial phase, the mask was too loose and the effect of tightness on the intensity of the sensors was the same. However, as the mask started fitting on the face, the effect of tightness on the intensity of the sensor changed because of the face structure and the mask composition, which resulted in the different readings of sensors at the same length and position inside the mask.

For the second experiment, a fabric mask was used with the strap length set to the loosest setting in a similar way to the surgical mask. Table 3 contains the results of the experiment with the light intensity for the left and right sensors recorded at each tightness step. The first reading was measured when the length of the strap was at the loosest setting at 19.25 cm, which provides the reading of intensities of 8 for the right and left sides of the mask, respectively. By progressing the experiment, the intensity of light started decreasing, and the tightness of the mask was achieved at the light intensity of 0 for the fabric mask. Based on the outcome, it can be stated that a fabric mask is better at providing protection against contamination than a surgical mask.

The intensity of light recorded by light intensity sensors within the mask is also directly proportional to the length of the strap, providing a comparable result to that of a surgical mask. When the length of the strap approaches 0 cm, the reading of both sensors drops to 0, indicating that the mask is tight according to the user. The key difference between surgical and fabric masks is the type of material used which resulted in the difference in intensity variations when compared to each at the same strap length.

For the third experiment, a KN95 mask was used, and the experiment process was repeated in a similar procedure as with surgical and fabric masks. The results of the experiment are displayed in Table 4 with the intensity of the light recorded at each tightness step for the left and right sensors. The different structure of the mask makes it unique and more effective than the other masks. A similar setup of tightness was made for the KN95 mask, but because of its uniqueness, some modifications were made in the strap length in order to perform the experiment; the length of the strap has its loosest setting at 25 cm and was tightened by 1 cm each step over time until it reached its tightest setting, which is 18 cm. At the loosest setting of 25 cm, the light intensities were measured at 7 for the left and right sides of the mask. The rate of variation of sensor light intensity strictly decreases compared to other masks when the strap is tightened.

These results indicate that the intensity of light recorded by light intensity sensors within the KN95 mask is exactly proportional to the length of the strap, implying that as the length reduces, so does the distance between the mask and the user’s face. It should be noted that the length of the strap on the KN95 mask varies from 18 cm to 25 cm, but the length on surgical and fabric masks varies from 0 cm to 19.25 cm. Because of the unique construction of N95 masks, when the length of the strap reaches 19 cm, the reading of both sensors drops to zero, indicating that the mask is tight and closest to the user’s face. Furthermore, when the length of the mask’s strap is reduced further by 1 cm, the intensity remains 0 but the mask feels tighter to the user.

**Figure 11:**
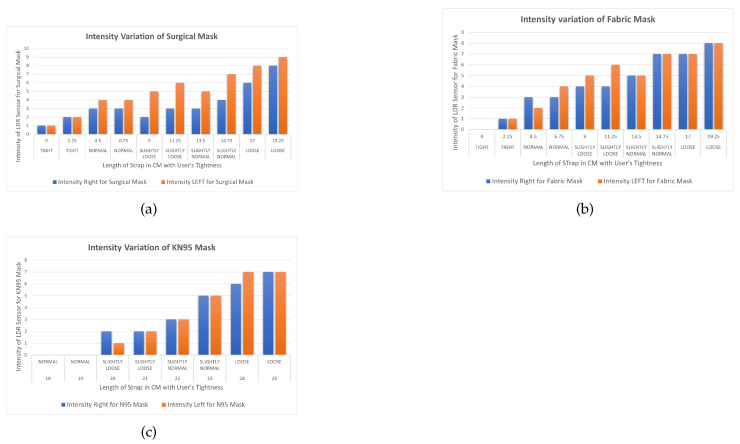
Variation of intensity with the length of strap with (**a**) surgical mask, (**b**) fabric mask, (**c**) KN95 mask.

To visualize the difference between the three different masks, Figure 11 provides a graph of these results, showing the correlation between the length of the strap, perceived user mask tightness, and light intensity from the left and right LDR sensors. After analyzing the findings of all three masks, it was determined that the intensity of light decreases with the length of the strap in all cases. It was also discovered that KN95 masks were superior to the other two masks since the minimal value of intensity was observed when the length was lowered to 0 in the surgical and fabric masks, but not in the KN95 mask. The intensity 0 in N95 masks was estimated when the length was 18 cm, indicating that these masks do not need to be tightened as much to provide better performance.

### 4.2. Variation of Temperature and Humidity with a Length of Strap of Surgical, Fabric, and KN95 Mask

The objective of this experiment was to determine the temperature and humidity inside the mask by varying the adjustable strap length using the same configuration as in the experiments with light-intensity sensors. Temperature and humidity indicate the inhalation and exhalation of the user and how it affects the fitness of the mask [27]. For this experiment, a DHT11 combined humidity and temperature sensor was added to the setup illustrated in Figure 9 directly facing the user’s mouth. As observed from the behavior of light intensity, a hypothesis was that when the strap length is tightened, the humidity and temperature value should increase proportionally. For these experiments, the process was the same as in Section 4.1. For each type of mask, the length of the strap was set to its loosest setting. Over time, this was tightened by 2.25 cm at each step until it reached its tightest setting. The experiments were repeated 10 times, each varying the strap length from 19.25 cm to 0 cm, and the results averaged.

Table 5 contains the measured temperature and humidity with a surgical mask. The first reading was measured when the length of the strap was kept at 19.25, which provides the reading of humidity and temperature of 58 and 25, respectively. The range of the sensor data varied from 0 to 50 degrees Celsius in temperature and 0–100% for humidity. As the mask was tightened, the values for temperature and humidity increased relatively proportionally. When the length of the strap was set to 0 cm, the values of the sensor reading were 90 and 36 for humidity and temperature, respectively.

**Figure 12:**
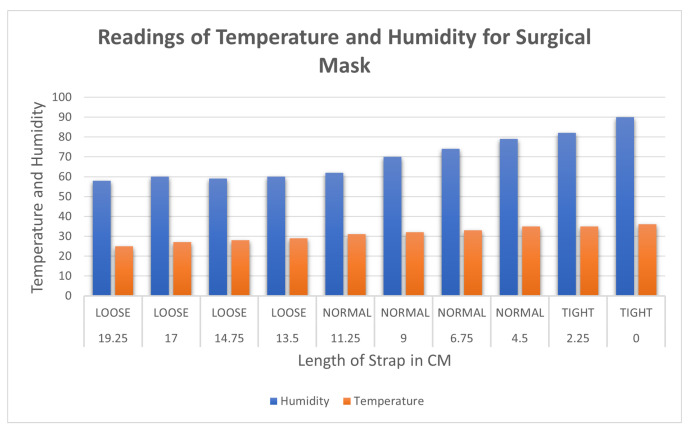
Variation of temperature in Celsius and humidity inside the surgical mask.

Figure 12 shows that the hypothesis was proven, as temperature and humidity follow a similar trend of increasing with the decrease in length of the strap. The rate of increase in values is inversely proportional to the length of the strap but directly proportional to the tightness. As the length of the strap decreases the tightness increases. The trend of the plot is increasing temperature and humidity as the mask is tightened.

The data for temperature and humidity for the fabric mask using the dht11 sensor are recorded in Table 6. The first reading was measured when the strap was kept at the loosest end, which is 58 and 25 for humidity and temperature, respectively. As the experiment progressed by decreasing the strap length, humidity and temperature increased. When the strap length was reduced to 0 cm, the maximum values for humidity and temperature recorded were 92 and 36, respectively.

**Figure 13:**
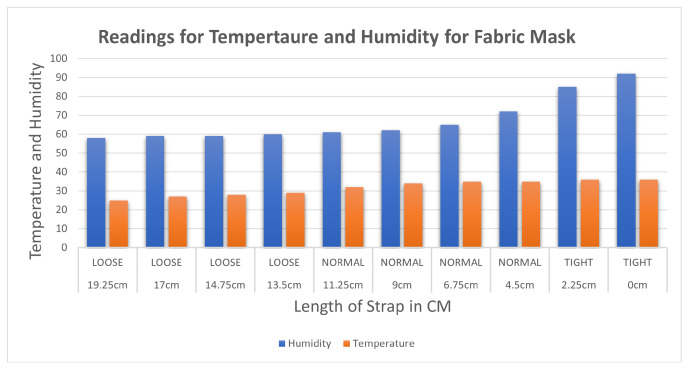
Variation of temperature in Celsius and humidity inside the fabric mask.

Figure 13 shows the similar trend of the increase in humidity and temperature with the decrease in the length of the strap. The rate of increase in values is inversely proportional to the length of the strap but directly proportional to the tightness. As the length of the strap decreases the tightness increases. The trend of the plot shows increasing temperature and humidity as the mask is tightened.

The results for the temperature and humidity sensor with the N95 are displayed in Table 7. At the loosest setting, the N95 mask reads a temperature of 28 and a humidity of 78. As the mask is tightened the trend is observed with the KN95 mask when the length of the strap is decreased with respect to the temperature and humidity.

**Figure 14:**
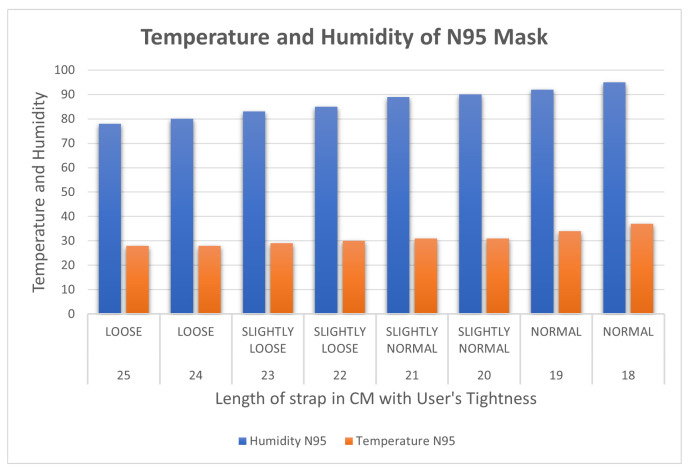
Variation of temperature in Celsius and humidity inside the KN95 mask.

Figure 14 shows that humidity, like temperature, increases as the length is decreased. After carefully evaluating the findings of both experiments, it was determined that the temperature and humidity in a mask rise as the strap length decreases. It was discovered that because of the restricted airflow in KN95 masks, temperature and humidity increased fast when the strap was tightened, making it better for protection than the surgical mask. Furthermore, it should be noted that the temperature and humidity detected are affected by other parameters, such as the mask’s quality/material and construction (in KN95).

Based on the outcome of the above experiments, it is possible to determine that the correlation between three types of masks and sensors impacts the mask’s fitness. The intensity of the light is inversely related to the tightness and fit of the mask, although temperature and humidity are directly proportional. This part focuses on tightening and fitting the mask utilizing the blueprint built using DC motors and gears to automate tightening and fitting.

### 4.3. Evaluating Mask Automatic Adjustment

**Figure 15:**
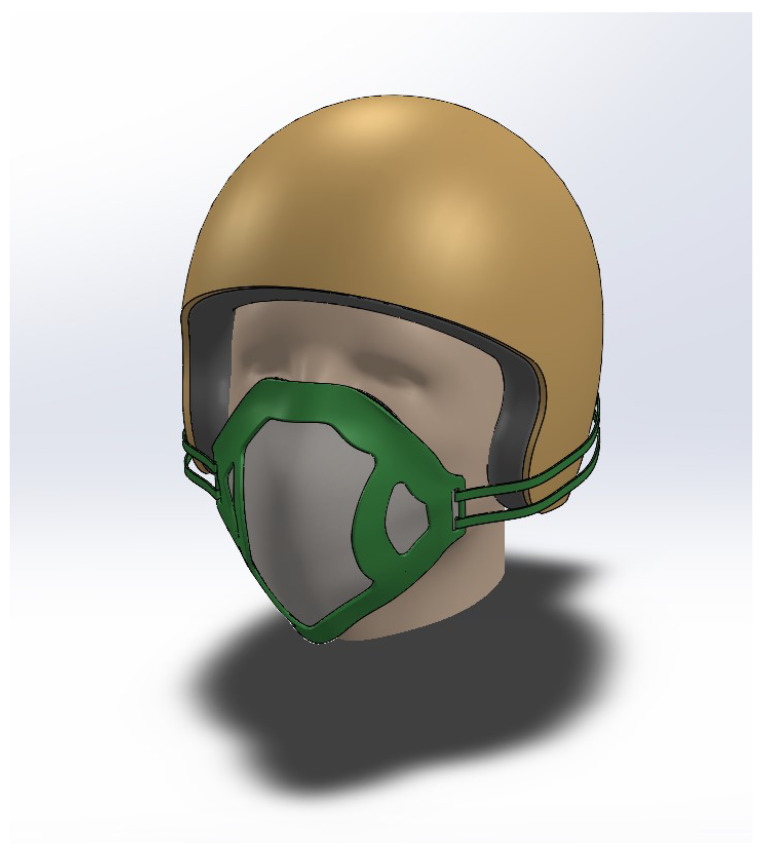
3D render of a person wearing the mask brace.

The objectives of these experiments were to assess the efficacy of the automatic mask adjustment mechanism. This mechanism utilizes four straps connected to a brace, with sensor information provided by four light intensity sensors. The data collected from these sensors were utilized to calculate the rotation of the DC motors attached to the mask straps. In this manner, the rotation value is dependent on the current light intensity value. The equipment employed to tighten the straps is a DC motor that operates on a PWM (pulse with modulation) pin on the microcontroller with a power range of 0–1024, where 0 represents no power and 1024 represents high power. Additionally, the LDR sensors are connected to an analog pin with a value range of 0–1024. Figure 15 illustrates the experimental setup, which comprises a 3D-printed mask brace placed over an N95 mask. The participant is instructed to sit in an idle position after donning the setup.

For each step, the value of the intensity of light was mapped with the intensity attribute. The power delivered to the motor at that intensity was computed with the tightness attribute as specified by the user. Both motors operate in a similar manner, utilizing the power series calculated in the table.

**Figure 16:**
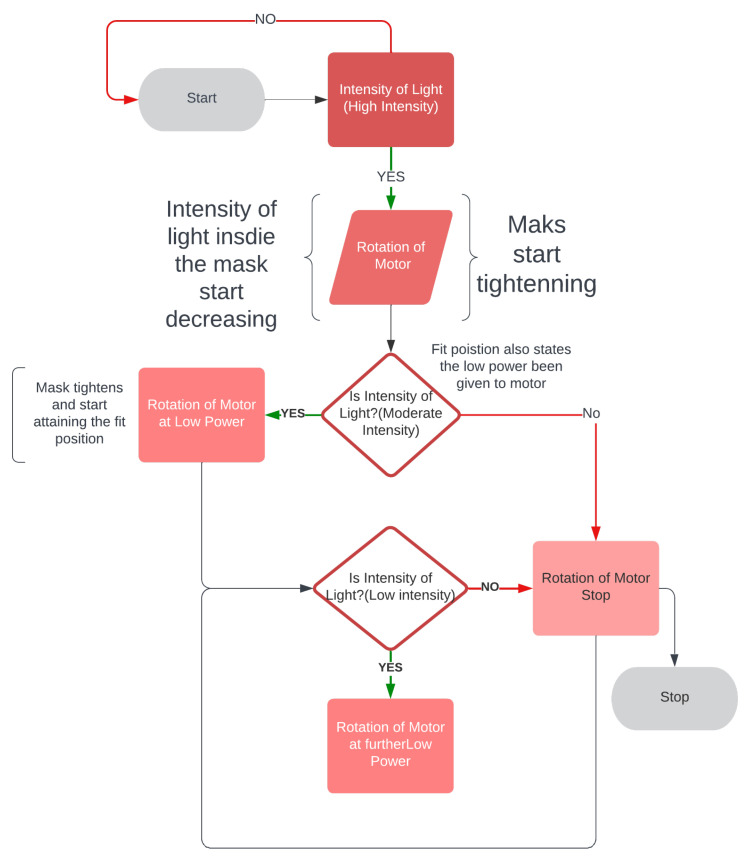
Algorithm flowchart.

The DC motors are connected with the Arduino and work as per Algorithm 1. When the mask brace is worn, the sensors start collecting the intensity of the light from the LDR sensors. When the motor is switched on it starts rotating depending on the initial average value coming from two LDR sensors per strap. With a delay of 500 milliseconds, the second value is based on which power is given to the DC motor. Temperature and humidity also affect the power. It reads the value of temperature and humidity at an interval of one second and checks if the value is increasing or decreasing based on the variation of power supply. In the algorithm designed, the light intensity energy is converted to power supply for the DC motor; the higher the intensity of light, the higher the power supply and the rotation of the motor. If the intensity of light is less, sensors will be able to detect the intensity, but not much power will be generated, which implies, as per the algorithm, that enough torque is required for rotating the motors. In short, more intensity of light is required. This also states that the mask is in a fit state. The flowchart of the algorithm design can be seen in Figure 16.
**Algorithm 1:** An algorithm for detecting the mask fitness.
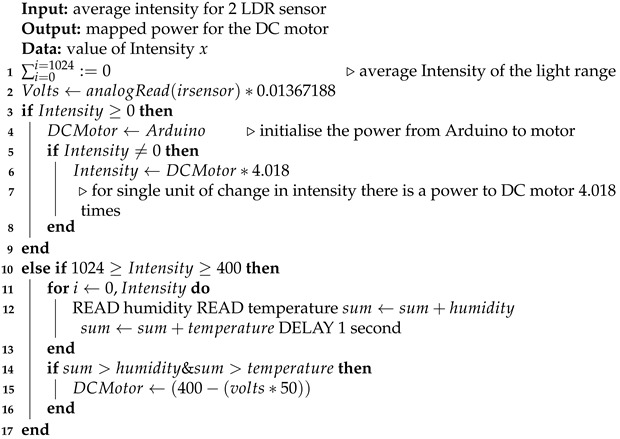


For these experiments, a user wears the mask brace using an N95 mask filter which is tied to the edges of the brace. The mask is set to its most loose setting and the automatic adjustment mechanism is activated. The adjustment mechanism algorithm takes control of the DC motors and tightens the mask straps until it reaches a state that it considers ideal.

#### 4.3.1. Motor Response to Light Intensity

To first investigate the response of the motor algorithm to the automatic tightening process, the light intensity, motor power, and light intensity were recorded. Table 8 lists the results of the experiment.

#### 4.3.2. Variation of Intensity of Light

The aim of this experiment was to investigate how light intensity varies with respect to the rotation of the motor which adjusts the mask straps attached to the mask brace. Understanding this behavior allows the creation of an optimal automatic mask adjustment algorithm. When the microprocessor is powered up, LDR sensors begin capturing the intensity of the light, and after some time, when the motor is turned on, an immediate spin in the motor is noticed, which begins tightening the strap of the brace. As a result, the mask brace is pulled closer to the user’s face, and the light intensity of the sensor gradually decreases, slowing the rotation. When the light intensity hits zero, the spinning of motor gears stops. The algorithm was developed to rotate the motor depending on the intensity of light such that for a single intensity of light there is rotation in the motor based on the amount of power which means if the intensity of light from the average of LDR sensors is 1 then there is a power from the DC motor but there is not enough torque to rotate the gears.

The observation of variation of intensity is divided into three steps. The procedure for the experiment is that the mask brace is put on; it does not fit and is a bit loose in the beginning. The average intensity of the upper two LDR sensors is computed with the rotation of power in the first stage, then the bottom two, and finally all of the sensors at their positions are turned on and the rotation of the motor is determined. Each graph is plotted on the axis of time and rotation of the motor, where x is time and the secondary y-axis is a rotation of the motor. The primary x-axis is a variable entity that varies with time and rotation. The variable entity in each graph is plotted with a logarithmic function below to show the skewness of the larger data of intensity temperature and humidity with the rotation of the motor in order to determine the percent change or factors that affect the motor rotation.

**Figure 17:**
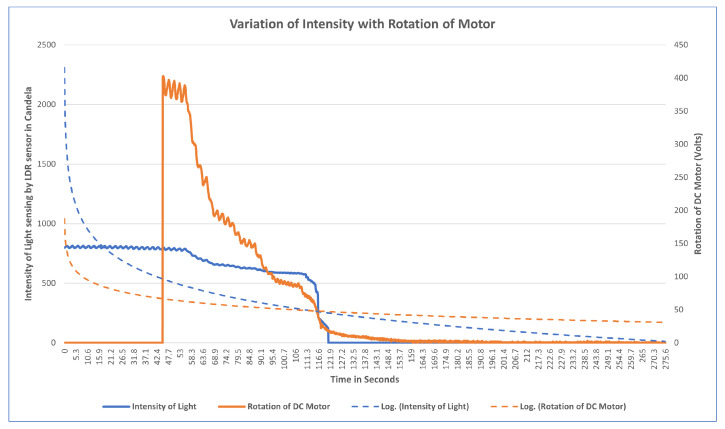
Variation of rotation of motor with the average intensity of light of upper LDR sensors.

**Figure 18:**
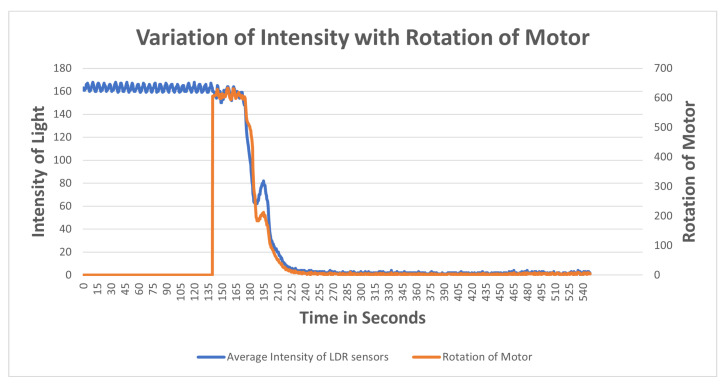
Variation of rotation of motor with the average intensity of light of bottom LDR sensors.

**Figure 19:**
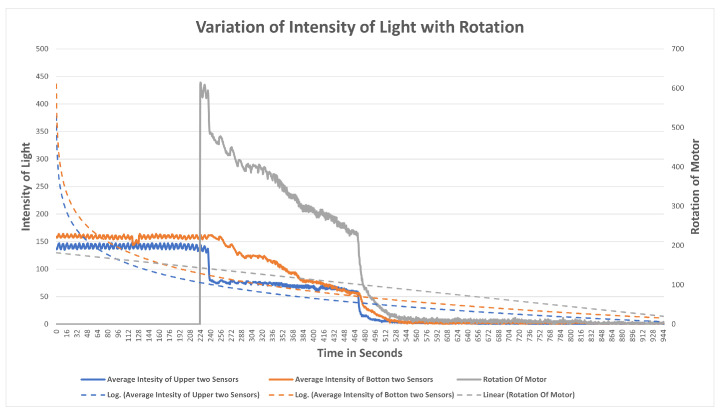
Variation of rotation of motor with the average intensity of light of all LDR sensors.

1. Observation for upper two sensors
  Figure 17 illustrates the results of an experiment when the mask was mounted, no power was supplied to the motors, as witnessed from time 0–42.4, the sensors measuring the intensity with no rotation. Motors were switched on from 42.4 to 47.4 and a light source was directed at sensors at the same time. When the intensity of light reaches 400, the rotation of the motor detected is 402, indicating that at higher intensities, there is maximum rotation. The intensity of light gradually decreases as the rotation slows down between 56 and 115 seconds. A progressive shift is noted at the time period of 115 s after which both intensity and rotation have a steady decline in values. At the interval of 121 seconds the intensity starts varying in the range of 0–1; at this interval, rotation becomes variable in the range of 0–10. Algorithm 1 is being satisfied here. From the plot it is observed that as the length of the strap decreases the average intensity of LDR sensors decreases which also meets the hypothesis in Section 4.1 that tightness is inversely proportional to the length of the strap and it increases with the decrease in intensity. It can also be concluded that the rotation of the motor is directly proportional to the intensity of light.
2. Observation for bottom two sensors
  The aim of this experiment was to investigate the impact on the bottom LDR sensors during the mask adjustment process. The experiment is performed in the same way as for the upper two sensors. The bottom sensor starts sensing the intensity while the motor was off. From Figure 18 it can be observed that the variation of average intensity of the bottom two LDR sensors is different to that of the average of the upper sensors and to the rotation of the motor. When the motor is turned on it takes some time to process the intensity and then starts decreasing gradually. It is observed that the rotation of the motor is directly proportional to the average intensity sensed by the bottom LDR sensors. The rate of variation as compared to the upper two sensors is significantly distinct because of the position of the sensors and the face structure. The bottom two sensors cover the area of the face near to the chin and jaw line. The position of the source of light is always directed towards the face from the ceiling, which in this case results in low/less light reaching the bottom two sensors. This results in less intensity captured and lowers the speed of rotation as compared to the upper two sensors.
3. Observation of All Sensors
  When all the sensors are put to working in a similar scenario, Figure 19 is obtained, in which the grey line indicates the rotation of the motor, the blue line indicates the average intensity of the upper two sensors, and the orange line indicates the average intensity of the bottom two sensors. The behavior of the graph is mocking the same variation as that of the above scenarios, which is that average intensity is directly proportional to the rotation of the motor with a multiple of 4.081. The graph depicts a clear picture of variation when the motor is turned on; in a few seconds there is a gradual decrease in the intensity of the LDR sensor and it is strictly decreasing with a decrease in the intensity. The resulting plot satisfies the behavior of all the upper and the bottom sensor processes with the rotation of gears and the DC motor.

#### 4.3.3. Variation of Temperature and Humidity Inside the Mask

**Figure 20:**
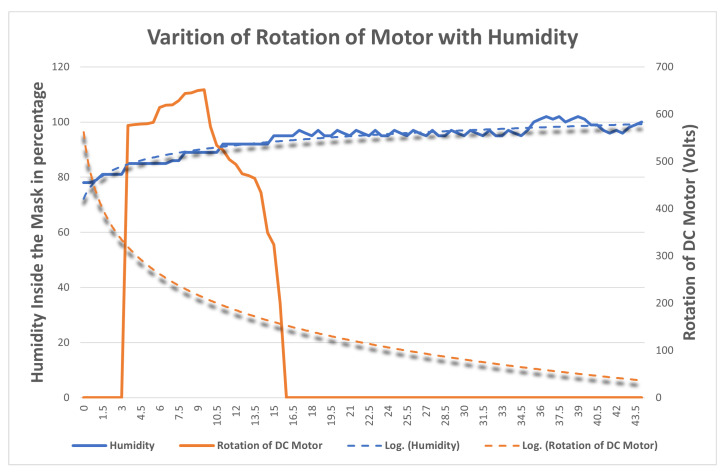
Variation of rotation of motor with humidity inside the mask.

**Figure 21:**
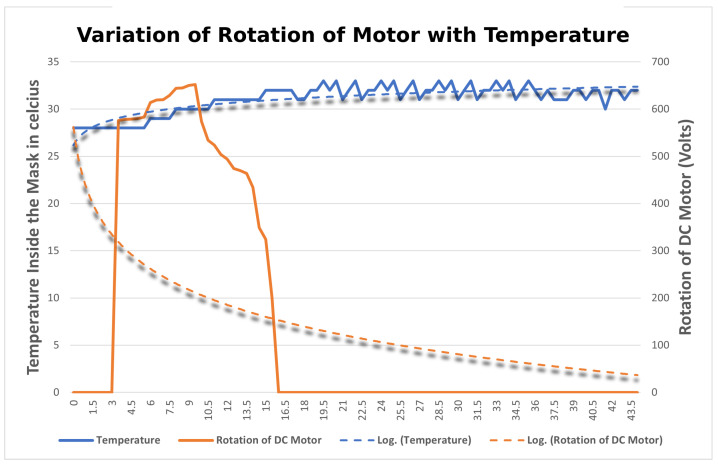
Variation of rotation of motor with the temperature inside the mask.

The aim is to find the variation of temperature and humidity of the DHT11 sensors inside by changing the length of the string with the DC Motor. The DHT11 sensor is fitted inside the cavity of the filter attached to the mask brace. The setup is similar to that in Section 4.3.2. The sensor collects the temperature and humidity readings at different lengths of string and their readings are compared and categorized with the rotation of the motor. The variation of temperature and humidity sensor is plotted onto a graph in Figure 20 and Figure 21, respectively. When the motor starts rotating there is a gradual increase in the temperature and humidity observed inside the mask. The blue line in the graph indicates the temperature and humidity and the orange line indicates the rotation of the motor. When the temperature and humidity start varying in the range of 90–100, the rotation of the motor starts decreasing. After analyzing the results, it was observed that the readings of the DHT11 sensor are quite different from that of the LDR sensor. As the length of the strap is tightened, the temperature and humidity recorded by the sensor located on the inner side vary inversely with the rotation of the motor. This is because the length of the string decreases and the mask is fitting to the user’s face as a result of the humidity inside the mask increasing. Thus, it can be concluded that the mask is tight for the user if the temperature and humidity are maximum inside, which also implies that the mask is a perfect fit for the user.

#### 4.3.4. Variation of Humidity Inside and Outside

**Figure 22:**
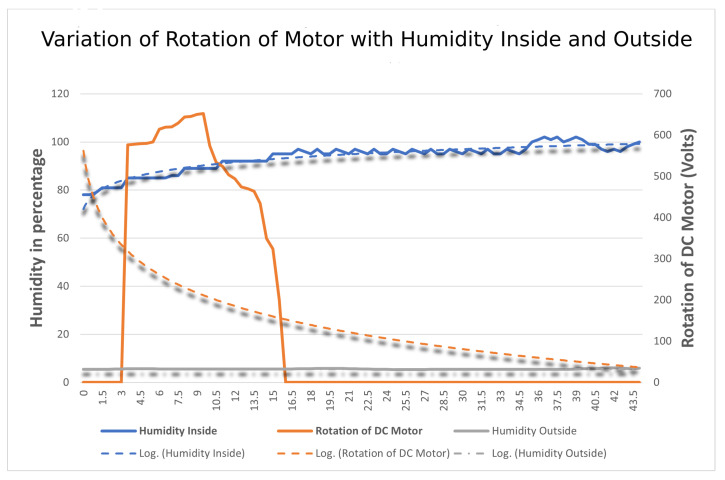
Variation of rotation of motor with the humidity inside and outside the mask.

The aim is to find the comparison of humidity of DHT11 sensors both outside and inside by changing the length of the string with the rotation of the DC Motor. In this, a total of two DHT11 sensors are used, one inside and the other outside, retrofitted in the middle of the mask. The setup is similar to that in Section 4.3.3. All the sensors collect the humidity reading at different lengths of string and their readings are compared and categorized according to the sensor located in and out of the mask with the power given to the DC Motor in terms of rotation. The variation of humidity of sensors is plotted onto a graph in Figure 22 w.r.t to time at the X-axis, humidity, and rotation of motor recorded at the Y-axis. After analyzing the results, it was observed that the readings of the DHT11 sensor outside of the masks were quite different from the one present inside. As the length of the strap is tightened, humidity recorded by the sensor located on the inner side varies inversely with the humidity recorded by the sensor located outside.

#### 4.3.5. Variation of Temperature Inside and Outside

**Figure 23:**
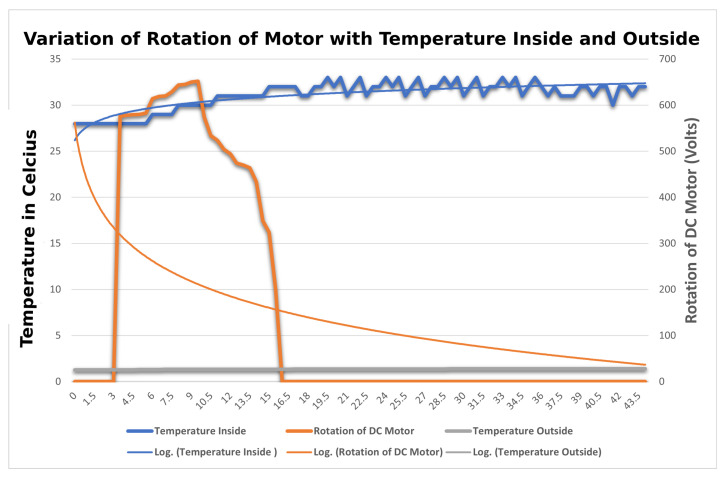
Variation of rotation of motor with the temperature inside and outside the mask.

The aim is to find the comparison of the temperature of DHT11 sensors both outside and inside by changing the length of the string. In this, a total of two DHT11 sensors are used, one inside and the other outside, retrofitted in the middle of the mask. The setup is similar to that in Section 4.3.4. All the sensors collect the temperature readings at different lengths of string and their readings are compared and categorized according to the sensor located in and out of the mask with the power given to the DC Motor in terms of rotation. The variation of temperatures of sensors is plotted onto a graph in Figure 23 w.r.t to time at the X-axis, temperature, and rotation of motor recorded at Y-axis. After analyzing the results, it was observed that the readings of the DHT11 sensor outside of the masks were quite different from the ones present inside. As the length of the strap is tightened, the temperature recorded by the sensor located on the inner side varied inversely with the temperature recorded by the sensor located on the outside.

## 5. Results and Discussion

The primary outcome of the study was the examination of the mask brace IoT technology under various conditions. The technology was subjected to multiple trials to collect data and evaluate the fluctuation of tightness in relation to factors such as light, temperature, and humidity. The proposed technology aims to offer a fundamental yet salient layer of defense against viral and contaminant exposure for the user.

When three different commercially available masks are evaluated on the basis of improved protection, as shown in Figure 11, the intensities vary in direct proportion to light, but the rate of increase differs. The intensity of the light detected by the sensors increases as the mask loosens. The length of the strap is then compared to the intensity measured by the sensors, which is inversely proportional.

**Figure 24:**
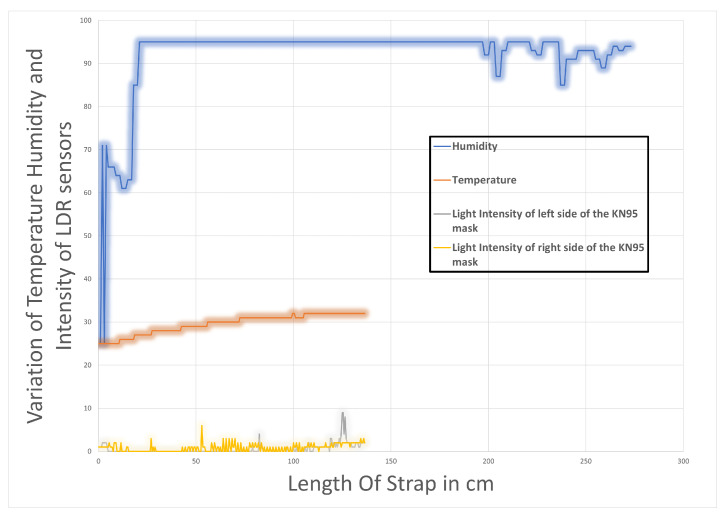
Data collected after the mask is worn for 2 min.

The KN95 mask is then selected above the other masks and retrofitted with a DHT11 sensor. The user wears the mask for two minutes while conducting tasks such as speaking, walking, and more, and can be seen in Figure 24. Figure 3 depicts the interior side of the mask; while carrying out the studies, the location of the sensors was also taken into consideration and altered as needed to avoid bias in the tests, as it was discovered that if sensors were placed in any position other than the arrangement shown above, the data would be inaccurate. So, for the position of the DHT11 sensor, LDR sensor readings were acquired at several points inside the mask, and the optimal position where the intensity is zero was chosen.

The temperature gradually increased from the time the mask was put on until it was removed. The orange line denotes a rise in temperature. The blue line indicates that humidity increased sharply at first and then steadied after a few seconds of operation. The LDR sensors on the mask’s left and right sides behaved similarly. The movement generated by speaking pushed the mask away from the optimum fit position, causing an increase in the intensity measured by the LDR sensors, as seen by the grey and yellow lines in Figure 24.

The methodology employed in this study involved the selection of the most appropriate fit technique and commercially available mask out of three options. Subsequently, a series of experiments was conducted utilizing a 3D model mask brace to automate the tightening process. The results revealed that the variations observed for intensity and temperature/humidity were congruent with the observations made during manual strap adjustments to the mask. As illustrated in Figure 19, the relationship between the average of the two upper sensors and two bottom sensors with the rotation power was found to be indicative of the tightness and overall fit of the model. Initially, when no power was applied to the motor, the sensors were able to detect light within the model. However, as the motor was powered, a gradual decrease in the average intensities of the sensors was observed. This decrease in intensity corresponded with a decrease in power output from the motor, thereby demonstrating a direct correlation between intensity and rotation power. The algorithm and the 3D model were found to be effective in sensing the light inside the mask and adjusting the tightness to provide the best fit for the user. A tight and well-fitting mask is characterized by the absence of light inside the mask.

Limitations of the approach:

1. Scope
  The prototype was made targeting healthcare workers but is not limited to the general public with some modifications. Currently, the manuscript is focused on a technical proof of concept, but with ethics, approval, and testing, the proof of concept with different face structures and with different age groups will provide a different path for the research.
2. Cost
  Proof of concept was built using 3D printers and materials like a rubber motor which helps the gears to rotate. All jumper wires and physical sensors make the product expensive, which can be overcome by designing a printed circuit wearable sensor that will also resolve the issue of the heaviness of the product as they are lightweight and cheap.
3. Quality and Benefits
  One limitation of the working principle of the mask is that when wearing the mask initially the person must be in a space where the light is sufficient enough so that sensors can start sensing the intensity or else there will be a time-lapse of a few seconds before the motor starts.

The study mainly contributes to improving the fit of the mask and providing the best available measured technique using light sensors to analyze the tightness and adjust it without any effort from the user and with the help of temperature and humidity sensors to track the data of the user’s breathing. The main aim of the technique and approach used is to provide the best fit of the mask on the face and to retain that fit by tightening using motors and gears.

## 6. Conclusions and Future Work

Before the COVID-19 pandemic, face masks were used at healthcare institutions or in roles where they were essential. A doctor may recommend it to limit the flu or the spread of diseases. However, since the notion was inverted, there is no longer a job requirement or prescription to wear a mask to limit the spread of the COVID-19 virus. It becomes vital for our health, as well as the health of our loved ones and the environment. Face masks protect the user by covering the user’s peripherals, such as the mouth and nose, with different layers certified by a health professional. However, if that layer has a broken seal, it is unlikely that masks will constantly remain in contact with the user’s face when doing activities.

This research aims to create a mask brace that uses IoT sensors and adjustable straps to determine the fitness of the user’s face. The best fit of the mask on the face provides protection against contamination and increases the resistance of the face mask against viruses. Sensors linked to the mask detect light breaches; the mask’s functionality is dependent on the intensity of the light. The light inside the mask indicates that the mask is not correctly fitted to the user’s face. The temperature and humidity sensor inside the mask is the second factor determining the fit of the mask. When there is no light inside the mask, both temperature and humidity are high and strictly increase to a specific level; however, when there is light, the humidity and temperature values continue to rise but at a slower rate.

Many experiments were carried out with various masks and sensor positions and compared to observe how the intensity, temperature, and humidity vary. If the sensor inside the mask does not sense the light intensity then it is considered the optimum fit for the user. The temperature and humidity rise when the user adjusts the display from the loose to the tight position. It states that whereas the mask changes to the ideal fit, there will be a progressive increase in temperature and humidity. Moreover, the algorithm designed for the mask is based on the average of two sensors at a time. Collecting the data from each individual sensor and redesigning the algorithm according to it can increase the accuracy for achieving the best fit.

Future planned work includes the use of a further array of sensors on the edges of the mask brace. Further investigation will be performed on the different positions of sensors. Using more precise gears will produce less heat and minimize the loss of rotation power. Finally, the plan is to investigate the use of new materials such as materials that can shrink and tighten with changes in electric current. Investigating these technologies can provide a richer and more precise mask adjustment result and work on automating the mask adjustment so that it can obtain the most excellent fit and notify the user if the mask’s optimal fit position is lost via a blinking LED or connecting with a mobile application. Various other approaches can be followed to make it more available to the general public, such as by making it lightweight.

## Figures and Tables

**Table 1:** Comparison of smart masks, their sensors, main focuses, and challenges.

Product	Project Hazel	CX-9	LG Wearable	Forcit	Air Pop	FaceBit
Ventilation	✓	✓	✓	N/A	N/A	N/A
Voice Modulation	✓	✓	✓	✓	N/A	N/A
Heavy	N/A	✓	✓	✓	✓	✓
Washable	N/A	N/A	N/A	✓	N/A	N/A
Sweat/Water Resistant	N/A	N/A	N/A	N/A	N/A	N/A
Data / Sensor fusion/ Sensing	N/A	N/A	N/A	N/A	✓	✓
Nano Technologies	N/A	N/A	N/A	N/A	N/A	✓
Automated Fitting	N/A	N/A	N/A	N/A	N/A	N/A
Notifying Filters	N/A	N/A	N/A	✓	✓	✓
Heavy Breathing/ Running	✓	✓	✓	N/A	N/A	✓
Cross Contamination	N/A	✓	✓	✓	✓	✓
Education Compliance	N/A	N/A	N/A	N/A	N/A	N/A
Comfort	✓	✓	✓	✓	✓	✓
Style/Fashion	✓	✓	✓	✓	✓	N/A

**Table 2:** Readings of the experiment of light intensity sensors with surgical mask.

Length of Strap in cm	Tightness	Intensity Left	Intensity Right
19.25 cm	LOOSE	9	8
17 cm	LOOSE	8	6
14.75 cm	SLIGHTLY LOOSE	7	4
13.5 cm	SLIGHTLY LOOSE	5	3
11.25 cm	SLIGHTLY NORMAL	6	3
9 cm	SLIGHTLY NORMAL	5	2
6.75 cm	NORMAL	4	3
4.5 cm	NORMAL	4	3
2.25 cm	TIGHT	2	2
0 cm	TIGHT	1	1

**Table 3:** Readings of the experiment of light intensity sensors with fabric mask.

Length of Strap in cm	Tightness	Intensity Left	Intensity Right
19.25 cm	LOOSE	8	8
17 cm	LOOSE	7	7
14.75 cm	SLIGHTLY LOOSE	7	7
13.5 cm	SLIGHTLY LOOSE	5	5
11.25 cm	SLIGHTLY NORMAL	6	4
9 cm	SLIGHTLY NORMAL	5	4
6.75 cm	NORMAL	4	3
4.5 cm	NORMAL	2	3
2.25 cm	TIGHT	1	1
0 cm	TIGHT	0	0

**Table 4:** Readings of the experiment of light intensity sensors with KN95 mask.

Length of Strap in cm	Tightness	Intensity Left	Intensity Right
25 cm	LOOSE	7	7
24 cm	LOOSE	7	6
23 cm	SLIGHTLY NORMAL	5	5
22 cm	SLIGHTLY NORMAL	3	3
21 cm	SLIGHTLY LOOSE	2	2
20 cm	SLIGHTLY LOOSE	1	2
19 cm	TIGHT	0	0
18 cm	VERY TIGHT	0	0

**Table 5:** Temperature and humidity with varied tightness with a surgical mask.

Length of Strap in cm	Tightness	Humidity	Temperature
19.25 cm	LOOSE	58	25
17 cm	LOOSE	60	27
14.75 cm	LOOSE	59	28
13.5 cm	LOOSE	60	29
11.25 cm	NORMAL	62	31
9 cm	NORMAL	70	32
6.75 cm	NORMAL	74	33
4.5 cm	NORMAL	79	35
2.25 cm	TIGHT	82	35
0 cm	TIGHT	90	36

**Table 6:** Temperature and humidity with varied tightness with a fabric mask.

Length of Strap in cm	Tightness	Humidity	Temperature
19.25 cm	LOOSE	58	25
17 cm	LOOSE	59	27
14.75 cm	LOOSE	59	28
13.5 cm	LOOSE	60	29
11.25 cm	NORMAL	61	32
9 cm	NORMAL	62	34
6.75 cm	NORMAL	65	35
4.5 cm	NORMAL	72	35
2.25 cm	TIGHT	85	36
0 cm	TIGHT	92	36

**Table 7:** Temperature and humidity with varied tightness with a KN95 mask.

Length of Strap in cm	Tightness	Humidity N95	Temperature N95
25 cm	LOOSE	78	28
24 cm	LOOSE	80	28
23 cm	SLIGHTLY LOOSE	83	29
22 cm	SLIGHTLY LOOSE	85	30
21 cm	SLIGHTLY NORMAL	89	31
20 cm	SLIGHTLY NORMAL	90	31
19 cm	NORMAL	92	34
18 cm	NORMAL	95	37

**Table 8:** Mapping of the intensity to the power of rotation of DC motor.

Light Intensity	Light Intensity Attribute	Power Given to Motor	Tightness Attribute
0	dark	0	very Tight
1	little dark	0	tight
2	normal	0	less tight
3	normal	0	less tight
4	room light	0	little loose
5	room light	0	loose
6	max room light	50	loose
7	bright	100	very loose
8	bright	150	very loose
9	bright	200	very loose
10	very bright	250	very loose
11	very bright	300	very loose
12	very bright	350	very loose
13	very bright	400	very loose
14	very bright	1024	very loose

## Data Availability

The data presented in this study are available on request from the corresponding author.
